# Exploring Health Professionals’ Knowledge and Attitudes on Eating Disorders

**DOI:** 10.1192/j.eurpsy.2025.1388

**Published:** 2025-08-26

**Authors:** E. Barone, L. Marone, V. Ruzzi, A. M. Monteleone

**Affiliations:** 1 Psychiatry, University of Campania “L. Vanvitelli”, Naples, Italy

## Abstract

**Introduction:**

Non-mental health professionals play an important role in the diagnosis and treatment of Eating Disorders (EDs).

**Objectives:**

This study aimed to explore attitudes toward EDs and knowledge of diagnosis, aethiology, and management of EDs among health professionals.

**Methods:**

A new questionnaire was validated and administered to residents and consultants working in disciplines involved in the management of EDs. Health professionals were grouped in the following areas: internal medicine, general practitioners, psychiatric area, psychological area, and surgical area. One-way ANCOVA and chi-square tests were employed to compare knowledge and attitudes among the study groups.

**Results:**

For all health professionals, the most deficient area was the aetiopathogenesis, while the best one was the management of physical complications. A gap in the knowledge of diagnosis, aetiopathogenesis and treatment emerged in nonmental health professionals. Psychotherapy effectiveness and the role of family members in the therapeutic process were not sufficiently acknowledged, and general psychological factors contributing to the onset of EDs were not recognized. Stigma was found primarily among surgeons, although all nonmental health professionals often considered those patients responsible for their ED.

**Conclusions:**

Inadequate knowledge and impaired attitudes toward EDs occurr among health professionals. This type of stigma may impair early diagnosis and treatment of EDs. Educational programs should provide continuous education to update and improve the knowledge of EDs among non-mental health professionals.

**Disclosure of Interest:**

None Declared

